# ENGAGEMENT MATTERS TO OPTIMIZE COGNITIVE BENEFITS OF COGNITIVELY IMPAIRED PEOPLE IN COGNITIVE STIMULATION THERAPY

**DOI:** 10.1093/geroni/igz038.421

**Published:** 2019-11-08

**Authors:** Yan, Anna Zhang, Gloria H Y Wong, Terry Lum

**Affiliations:** 1 The University of Hong Kong, Hong Kong, China, Hong Kong; 2 The University of Hong Kong, Hong Kong, Hong Kong; 3 Department of Social Work and Social Administration, PokFuLam, Hong Kong

## Abstract

Background Cognitive stimulation therapy (CST), a 14-session themed groupwork for people with cognitive impairment, shows effectiveness in maintaining cognitive functioning, quality of life and communication. However, its mechanism of optimizing individual cognitive benefits is little known. Engagement, a state of being occupied by meaningful external stimuli, may be an overlooked link. Individual constructive engagement is defined as the verbal or motor individual behavior exhibited for the meaningful purposed activities. Objective To investigate the individual experience of engagement in CST and its effect on cognitive benefits. Methods A total of 108 participants were recruited from 8 community centers, 2 daycare centers and 2 residential care units in Hong Kong. Trained assessors not involved in CST delivery conducted the pre-and-post assessments using Alzheimer’s Disease Assessment Scale-Cognitive Subscale (ADAS-Cog). Trained raters observed each participant’s engagement under six 5-minute observation windows in total during CST sessions by time sampling using the adapted Myers Institute Engagement Scale. Results Paired t-test shows significant improvement in ADAS-Cog (Pretest: M=22.71, SD=12.63; Posttest: M=19.06, SD=10.93; MD=3.65, SD=7.38, t=5.138, p<.000). Individual constructive engagement was exhibited 23.3% of activity time averagely. It correlated with the change in ADAS-Cog positively(r=.292, p<.002). Greater individual constructive engagement predicted lower post-intervention ADAS-Cog score (B=-14.98, β=-.192, p<.001), indicating a better cognitive functioning, after controlling baseline ADAS-Cog score (B=.707, β=.816, p<.000) in the multiple linear regression analysis (R2 = .698, F (2,105) =121.38, p<.000). Conclusion Engagement is the potential mechanism to maximize individual cognitive benefits. Future studies can investigate contributing factors of engagement to improve intervention effectiveness

